# ﻿Two new species in the green lacewing genus *Santocellus* (Neuroptera, Chrysopidae, Leucochrysini)

**DOI:** 10.3897/zookeys.1226.140386

**Published:** 2025-02-07

**Authors:** Catherine A. Tauber

**Affiliations:** 1 Department of Entomology & Nematology, University of California, Davis, CA 95616 and Department of Entomology, Comstock Hall, Cornell University, Ithaca, NY, USA Cornell University Ithaca, NY United States of America

**Keywords:** Leucochrysine genera, morphology, Neotropical, patterns of variation, wing markings

## Abstract

Based on adult morphological features, two new species are described and assigned to the leucochrysine genus *Santocellus*: *Santocelluslegrandi***sp. nov.** from Central America and *Santocellushelene***sp. nov.** from Bolivia. As a result, the genus, which now contains five known species, becomes more securely defined. An updated catalog and an illustrated key to the known *Santocellus* species are provided.

## ﻿Introduction

The tribe Leucochrysini comprises a diverse and strikingly beautiful group of green lacewings (Neuroptera, Chrysopidae, Chrysopinae) (Brooks and Barnard 1990; Breitkreuz 2018). Currently, it contains seven genera, five of which are reasonably well characterized systematically – *Berchmansus* Navás, *Cacarulla* Navás, *Gonzaga* Navás, *Nuvol* Navás, and *Santocellus* Tauber & Albuquerque (Brooks and Barnard 1990; Tauber 2007, 2012; Tauber et al 2008; Sosa-Duque and Tauber 2023). All five of these genera are small (containing 1–6 species), and for three of them (*Berchmansus*, *Gonzaga*, *Santocellus*) the described generic features include a wide range of both adult and larval traits (Tauber 2012). However, with the exception of the virtually unknown monotypic genus *Neula*, the remaining large number of leucochrysine species are generally included in the ill-defined, large genus *Leucochrysa* McLachlan. The primary purpose of this short article is to describe two new *Santocellus* species. In doing so, the article provides information and new images for use in clarifying the pattern of morphological variation within the genus *Santocellus* and also for future comparison with species currently held in *Leucochrysa*.

### ﻿Dedication

The two new species described in this paper are named in memory of Jean Legrand (1944–2020), curator at the Muséum national d’Histoire naturelle, Paris (MNHN), and in honor of Jean’s wife, Hélène. Jean was an enthusiastic and productive systematist (primarily Odonata), an excellent and patient curator, and a delightful colleague and coauthor. See Pluot-Sigwalt and Perrin (2022) for a fine summary of his accomplishments and distinctive attributes. The following citations include Jean’s co-authored publications on the Neuroptera specimens under his care at the MNHN (Legrand et al. 2008; Tauber et al. 2017a, 2017b). Hélène Legrand, Jean’s wife of many years, is a medical doctor in Paris; she and Jean were welcoming hosts during my visits to the MNHN. I think of them both with great fondness.

## ﻿Materials and methods

The methods used here for preparing and studying the specimens were identical to those used in my previous studies of *Leucochrysini* (Tauber 2007, 2012; Tauber et al. 2008). The following abbreviations are used: **NHMUK**, Natural History Museum, London; **CAS**, California Academy of Sciences, San Francisco; **OUMNH**, Oxford University Museum of Natural History, Oxford; **SDMC**, San Diego Natural History Museum, San Diego.

### ﻿*Santocellus* generic features

Based on the shared features of the three *Santocellus* species described previously (see Tauber et al. 2008) and those described here, a significant set of shared characteristics appears and tentatively distinguishes adults of the genus from those in other leucochrysine genera. However, a word of caution is necessary. The range of variation in the above features is very poorly explored among the numerous *Leucochrysa* species. There may be considerable overlap.

The shared features include: bold markings on the forewings and body; a darkly colored frons; antennae longer than the forewing, but less than two times the length of the forewing; forewing with the first radial crossvein at or very near the origin of Rs; tarsi with distal segment dark, bearing a pair of elongate setae terminally, a pair of raised dorsal protuberances dorsally, and a distolateral pair of broadly extended tarsal claws each with a single spur. The male abdomen is slender; the terminus (S8+9) bears gonocristae along distoventral membranous margin and/or on a pair of tubercles slightly above the terminus. The male genitalia include a broad gonarcus bearing a prominent mesal beak and lateral lobes, but a tignum, gonapsis, parameres, quadrate hood, pseudopenis, and spinellae are lacking. The female genitalia include a round spermatheca and a sail-like velum, with a shallow invagination. The velum of the spermatheca appears to open to the bursa copulatrix directly; no duct appears to be present.

## ﻿Taxonomy

### ﻿Description of two new *Santocellus* species

#### ﻿ Santocellus legrandi sp. nov.

Taxon classificationAnimaliaNeuropteraChrysopidae

E596F489-F954-57B7-BC3D-D5E91CE47B2D

https://zoobank.org/A21C5BD5-27C7-419C-BAB1-5A0AC2BDDC58

Figs 1 , 2 , 3a , 4 , 5 , 6 , 8a, c, e , 10d , 11d , 12d

##### Etymology of name.

The species carries the surname of Jean Legrand (see Dedication above). Jean had a special love for art (especially painting); it is thus fitting that his name is associated with a beautiful lacewing that has stunning wing markings. The species name is a singular noun in the genitive case following a masculine genus name.

##### Type specimens.

**Figure 1.**
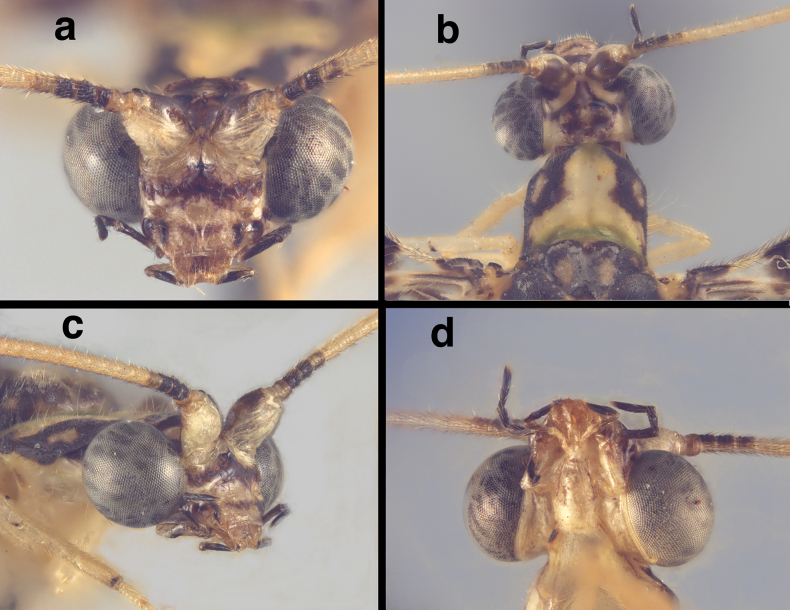
Head of *Santocelluslegrandi*, sp. nov. Holotype, male, Nicaragua, Río San Juan, Bartola (CAS) **a** frontal **b** head, prothorax, dorsal **c** head, frontolateral **d** ventral. Width across head (dorsal, including eyes), 1.85 mm.

**Figure 2.**
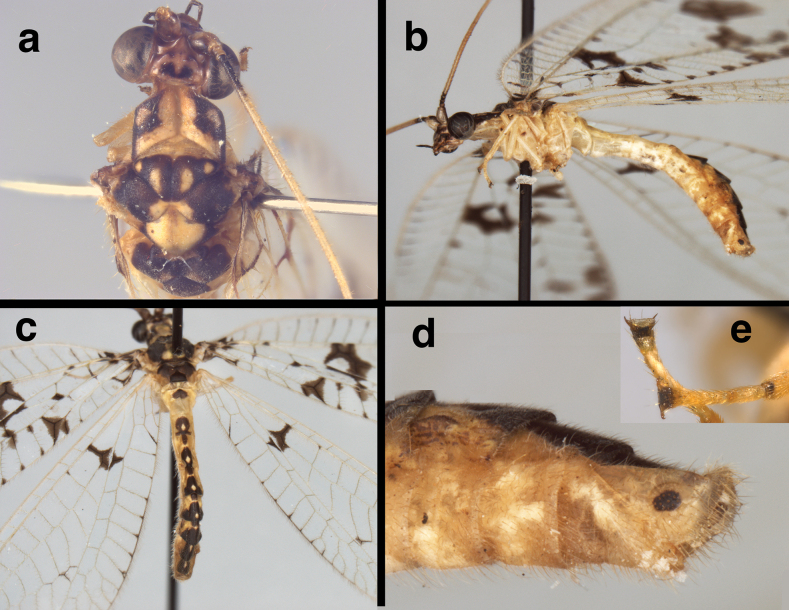
External features of *Santocelluslegrandi*, sp. nov. Holotype, male, Nicaragua, Río San Juan, Bartola (CAS) **a** head, thorax, dorsal **b** body, lateral **c** abdomen, dorsal **d** terminus, lateral **e** protarsal claw, lateral. Width across head (including eyes), 1.85 mm; diameter of callus cerci, ~0.2 mm.

**Figure 3.**
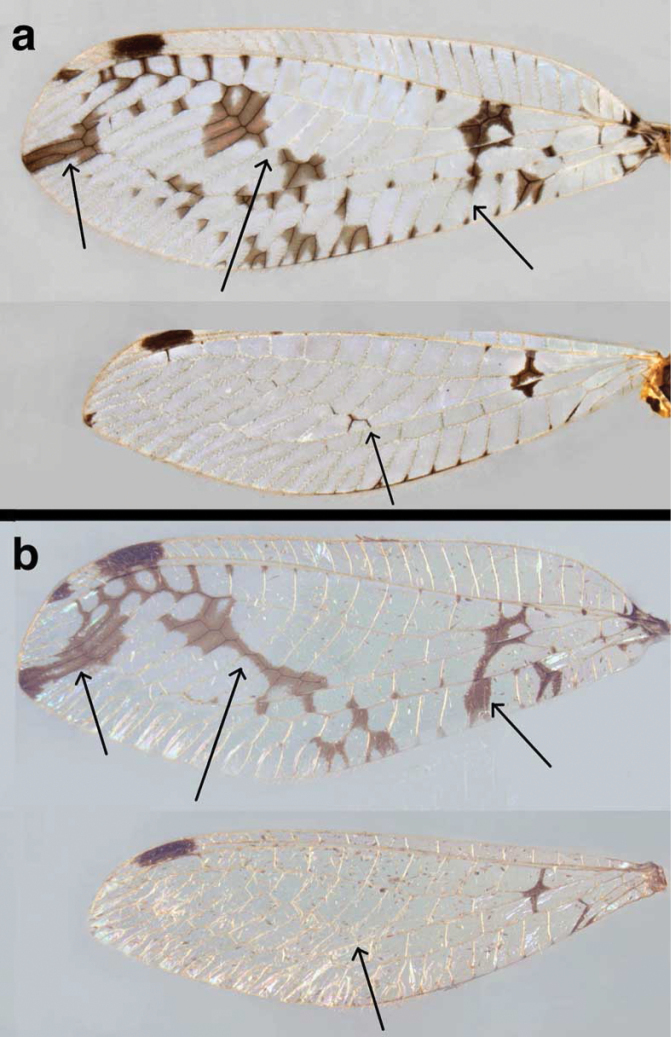
Wings of two new *Santocellus* species **a***S.legrandi* sp. nov., holotype, male, Nicaragua, Río San Juan, Bartola (CAS); forewing length, 18.7 mm **b***S.helene*, sp. nov., holotype, male, Bolivia, Cochabamba (CAS); forewing length, 19.4 mm. Arrows highlight interspecific differences in markings.

**Figure 4.**
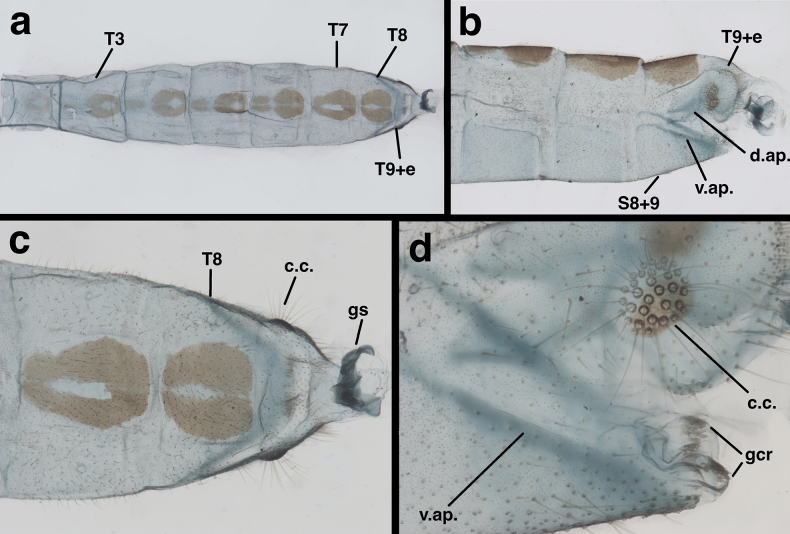
Male abdomen of *Santocelluslegrandi*, sp. nov., holotype, Nicaragua, Río San Juan, Bartola (CAS) **a** dorsum of abdominal segments A2-terminus **b** lateral view of abdominal segments A6-terminus **c** dorsum of abdominal segments A7 to terminus with gonarcus everted **d** lateral view of abdominal terminus. Length of T8 along dorsal margin, 0.68 mm. Abbreviations: c.c. callus cerci; d.ap. dorsal apodeme of T9+ectoproct; gcr field of gonocristae on terminal membrane of S9; gs gonarcus; S8+9 fused eighth and ninth sternites; T3, T7, T8 third, seventh, and eighth tergites; T9+e fused ninth tergite and ectoproct; v.ap. ventral apodeme of S8+9.

**Figure 5.**
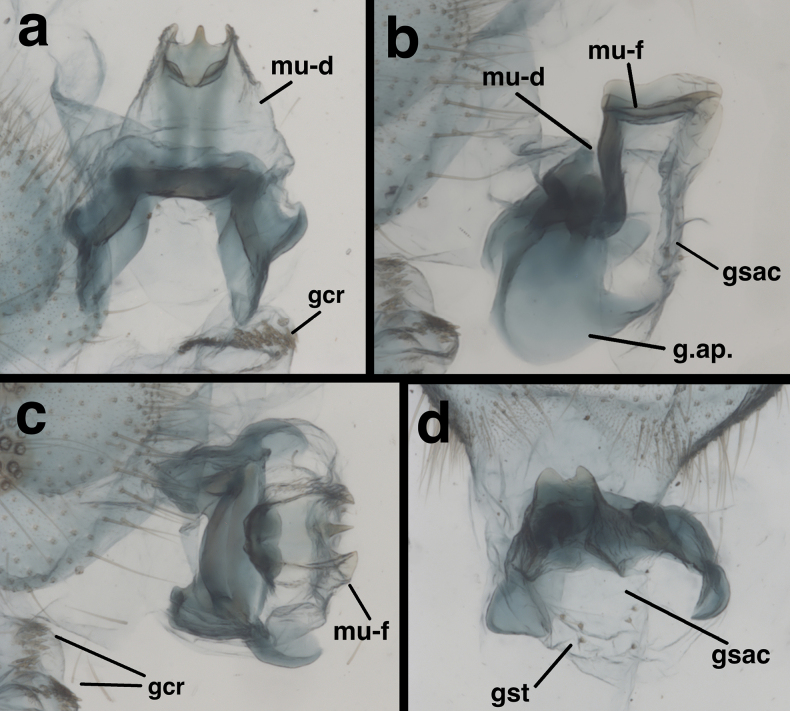
Male genitalia of *Santocelluslegrandi* sp. nov., holotype, Nicaragua, Río San Juan, Bartola (CAS) **a** gonarcal complex, dorsal **b** same, lateral **c** same, frontal **d** same, posteroventral. Abbreviations: gcr, field of gonocristae on terminal membrane of ninth sternite; gsac gonosaccus; gst gonosetae; g.ap. gonarcal apodeme; mu-d mediuncus, dorsal view; mu-f mediuncus.

**Figure 6.**
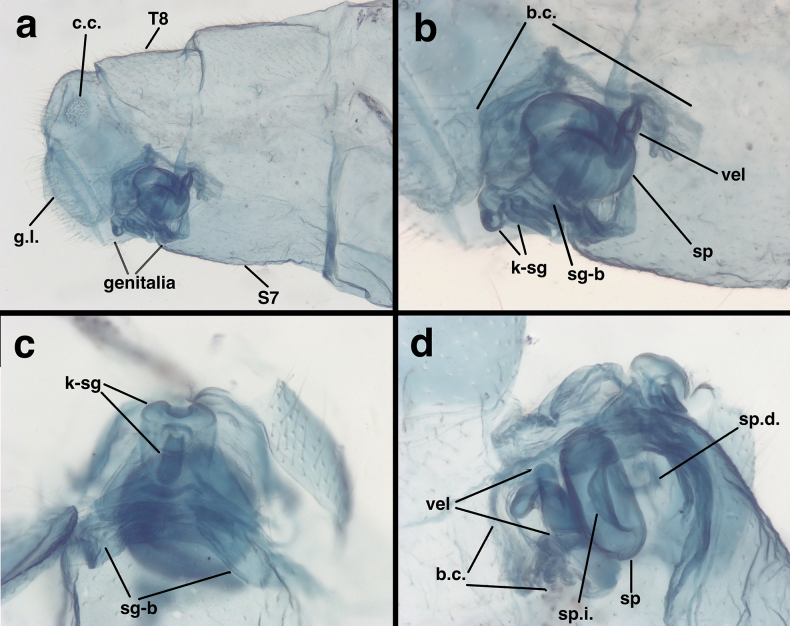
Cleared abdominal terminus of female *Santocelluslegrandi* sp. nov., allotype, Costa Rica, San José (SDMC) **a** segments A7 to terminus, lateral **b** genitalia, lateral **c** subgenitale, distal view **d** spermathecal complex below bursa copulatrix, lateral view. Abbreviations: b.c. bursa copulatrix; c.c. callus cerci; g.l. gonapophysis lateralis; k-sg distal knob of subgenitale; sg-b base of subgenitale; sp spermatheca; sp.d. spermathecal duct; sp.i. spermathecal invagination; S7 seventh sternite; T8 eighth tergite; vel, spermathecal velum.

**(sex and figures; verbatim label data [specimen location]) *Holotype*** • Male (Figs 1–5). – (1) Nicaragua, Rio San Juan / Bartola, 15 m, 12–15.10.09 / N 10 54 56/W84 17 57 / P. Duelli, U. & H. Aspoeck – (2) 11 [CAS]. ***Allotype*** • Female (Fig. 6). – (1) Costa Rica, 22 km / N. San Ysidro, / San Jose Prov. / V-24-85 – (2) J. T. Doyen, / P. A. Opler / Collectors [SDMC]. ***Paratype*** • Female. – (1) Belize: Cayo / Chiquibul Forest. Las Cuevas / Nature Trail / 31^st^ July 2001 / G. C. McGavin Coll. (2) G. C. McGavin Coll. / Pres. G. C. McGavin / OUM-2002-006 [OUMNH]. ***Paratype*** • Male. – (1) Honduras 1923 / Rio Paulaya: / Barraneo, iv-17 / 303 T. H. Haldell (2) Phillip A. Adams / Collection / 1998 bequest to / Calif. Acad. Sci. [CAS]. ***Paratype*** • Male. – (1) Chiriqui – (2) prob. *Leucochrysarisi* Esb-Pet, det. P. Adams ’80 [NHMUK; collection site probably in Panama; abdomen in poor condition; pieces of exoskeleton in attached micro-vial].

##### Description.

**Adult.** Medium-sized. ***Head*** (Fig. 1): Dorsum, including eyes 1.70 (F)–1.85 (M) mm wide; ratio of head width to eye width = 1.71 (M)–1.86 (F):1; sutures not visible. Vertex raised, with small posterior fold. Antenna long (28 mm, *n* = 1), ~1.5 times length of forewing; scape large, with approximately equal length and width; length of flagellomeres (beyond #5) ~1.6–1.8 times width, with setae in four rings; labrum emarginate. Torulus relatively large, extending slightly below mid-eye level. Frons with short to moderate length, pale setae, lower margin scalloped upward above clypeus (below toruli). Tentorial pits distinct, round, raised. Gena 0.24 mm long; ratio of genal length to distance between tentorial pits = 0.43:1. Clypeus quadrate, except dorsal margin broadly curved upward below frons, distal margin relatively straight, pale mesally, dorsal surface rounded, slightly raised mesally, with sparse, short to medium length amber setae. Labrum tapering anteriorly, with bilobed frontal margin, distinct cleft between lobes moderately deep, lateral margins tapering, long setae basally, shorter setae distally. Labium with submentum slender, rounded ventrally, with rounded, slightly bulbous tip. ***Coloration***: Markings on vertex, frons dark brown to black with tinge of deep red; broad, scalloped stripe across frons; vertex with large pentagonal mark, cream area within; clypeus with slender stripe across midsection, not reaching margins; labrum with dark reddish brown tinge mesally. Antenna with scapes pale frontally, dark brown dorsally, frontolaterally; pedicel cream with tinge of red distally; basal three flagellomeres dark brown to black frontally, medially; remaining flagellomeres pale. Maxilla with distal three palpomeres dark brown to black, basal two palpomeres pale with black markings laterally. Labium pale, with distal two palpomeres black, basal one pale.

**Figure 7.**
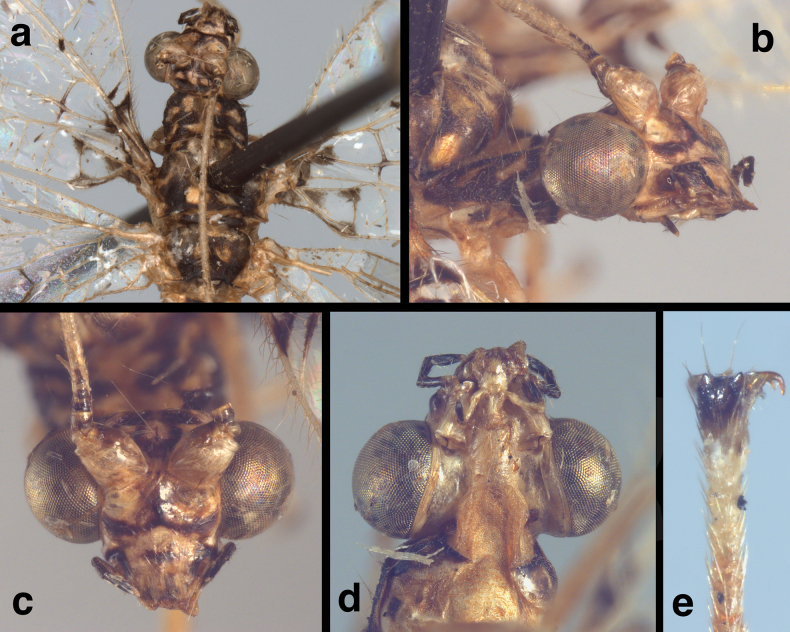
Head and thoracic region of *Santocellushelene* sp. nov., holotype, male, Bolivia, Cochabamba, Alto Palmar (CAS) **a** upper body (head, thorax) dorsal **b** head, prothorax, lateral **c** head, frontal **d** head, ventral **e** tarsus (dorsum) and tarsal claw (lateral). Width across head (including eyes) 1.82 mm.

***Thorax*** (Fig. 2a): Prothorax wider than long: 0.86–0.93 mm long, 1.11–1.27 mm wide, ratio of length to width = 0.69:0.78; setae scattered, long to medium length, thin, golden. Legs robust, of moderate length, with dense, short, golden setae; tarsal claws without basal dilation, but with two prongs. ***Coloration***: Pronotum cream mesally, dark brown laterally; anterior brown section broadly quadrate, extending about halfway across dorsal surface, with central section bearing cream spot; posterior section cream mesally, with brown stripe laterally. Mesonotum dark brown laterally, with patches of cream mesally on prescutum, scutum. Scutellum with large cream spot. Metanotum entirely dark brown dorsally. Thoracic pleuron, venter pale to light tan. Legs pale, mostly unmarked, with short to medium length, golden setae, few long, pale setae; terminal segment (base of tarsal claws) smooth, shiny, black dorsally, with pair of small (indistinct) swellings, two pairs of moderately long setae at tip (Fig. 7e). Tarsal claw moderately long, narrow distally, with single small spur; base slightly enlarged, without distinct dilation.

***Wings*** (Fig. 3a): Measurements: male left wing (*n* = 1–2); female, right wing (*n* = 1). Forewing: 17.3–18.7 mm long, 6.2–6.9 mm wide (maximum). Costal area moderately tall; tallest costal cell (#9, 10) 1.5–1.7 mm tall, 0.23–0.24 times maximum height of wing. First intramedian cell triangular, elongate, 1.5–1.6 mm wide, ~0.7–0.8 times width of third median cell. Origin of first radial crossvein ~0.5 mm distal to origin of Rs; radial area (between R and Rs) with single row of 13 or 14 closed cells; tallest radial cell 1.1–1.3 mm, 0.77–0.80 times taller than tallest costal crossvein. Three b cells, each full sized, slightly longer than tall; first b cell heavily marked on all sides. Two series of gradates; 8–10 inner gradates, 7 or 8 outer gradates; size of cells bounded by gradates fairly uniform, lengthening then shortening distally. Four to 5 b’ cells; last b’ cell wider than tall. Three intracubital cells, distal one open; first cubital crossvein located slightly basal to first mediocubital crossvein; icu1, icu2 each shorter than icu3; icu1+icu2 considerably longer than icu3. Vein 1A forked. Terminal margin with approximately 5 simple veinlets, 13 forked veinlets. Hindwing: 16.7–17.0.mm long, 5.0–5.1 mm wide. Eleven to 14 radial cells (counted from origin of R, not false origin); t cell absent. Two series of gradates; 5–9 inner gradates, 7 outer gradates. Two to 4 b cells; 4 or 5 b’ cells beyond im2. Terminal margin with 7 or 8 simple basal veinlets, 9–11 forked basal veinlets. ***Coloration***: Wing surface hyaline, with bold, dark brown markings. Forewing: Stigma with moderately large, dark brown spot basally, second, smaller dark brown spot distally; veins pale except when surrounded by dark marks: e.g., distal part of R and Rs dark brown with surrounding area dark brown; slightly elongate dark brown mark at tip of wing; two dark spots in approximate center of wing; scattered dark brown marks along basal 2/3 of lower edge of wing; veins dark when within dark spots. Hindwing: stigma with one large dark brown mark; base of RP, basal b cell dark, within dark brown mark; distal section of cu (upper fork) dark; posterior margin of wing with dark brown section, including suffusion at intersections with marginal veinlets.

***Male abdomen*** (Figs 4, 5). Segments 1–3 not particularly long or slender; distal segments only slightly larger than basal segments (male and female). Tergites shallow, with slightly rounded margins; each tergite with distinctive brown marking having white center spot. Tergites, sclerites, pleural region with numerous, short to medium length setae, microsetae. Abdominal sternites moderately tall (S6: ratio of length to width = 1.3:1), with dorsal margins straight, slanting, spiracles not enlarged, without microtholi; S9 with slightly long, robust setae distally, without microtholi. Callus cerci almost round (0.23–0.19 mm diameter), with 33–35 long trichobothria; cupuliform bases of variable size [those within central section larger (~0.026 mm) than those on periphery (~0.014 mm)]. Dorsum of T9+ectoproct rounded distally, fused mesally, with very small to no cleft; no midline suture visible; with long setae, densest along distal margin; dorsal apodeme simple, straight, short, extending around proximal side of callus cerci. S8+9 fused without suture, tapering to acute apex (lateral view); S9 slightly darker than S8; ventral apodeme extending across dorsal surface of S8+9; distal margin of S8+9 spatulate, with terminal membrane prominent; membrane bearing moderately heavy gonocristae in two broad (somewhat irregular) lateral patches, single, small mesal patch of several (~3) gonocristae. ***Coloration***: Dorsum of each segment pale with large black central mark; marks on T2–T8 with prominent white spot in center of black area; mark on T1 without white spot; venter, pleuron white to cream. Setae golden, long; dorsal half of callus cerci light cream colored; ventral half black.

***Male genitalia*.** Tignum, gonapsis, parameres, quadrate hood, pseudopenis, spinellae absent; S8+9 fused, elongate, not extending beyond T9+ectoproct. Gonarcus broad, with very small gonocornua on frontolateral margin of gonarcal bridge, with relatively large, rounded gonarcal arms expanded forward slightly; mediuncus in two sections; basal section extending from below gonarcal bridge, with pair of internal apodemes dorsomesally, with dorsal surface membranous, broad basally, tapering distally; distal section of mediuncus extending perpendicularly from tip of basal section, distally with flange-like lateral arms partially surrounding mesal beak; gonosaccus beneath mediuncus, stiff, flat, laterally with a few gonosetae on chalazae.

***Female abdomen*** (Fig. 6; *n* = 2, both slightly teneral). Callus cerci round, 0.14 mm in diameter, with 36–37 trichobothria of variable length; cupuliform bases also of variable diameter (0.010–0.029 mm). Dorsum of T9+ectoproct rounded posteriorly, tall (lateral view), extending approximately to lateral margin of gonapophysis lateralis. Sternite 7 with dorsal margin relatively straight; distal margin blunt, not particularly heavy, with setae mostly missing. Gonapophyses laterales slender, occupying approximately half of abdominal posterior margin; interior membranous area not greatly expanded or inflatable. T9+ectoproct truncate posteriorly, extending slightly below gonapophyses laterales (lateral view). Gonapophyses laterales occupying ~60–70% of posterior margin of abdomen.

***Female genitalia*.** Entire genital structure compact; spermathecal complex, bursal duct, base of subgenitale all contained below bursa copulatrix. Subgenitale broadly based, with distinct quadrate knob distally; distal section of knob bilobed, with broad, flat trough between lobes; lower frontal region of subgenitale with elongate, downward-projecting, snout-like protuberance (knob). Spermatheca round, doughnut shaped, with broad invagination, distal end with relatively large tapering, sclerotized, coiled and curved tubule (velum), with slit along most or full length, apparently opening to bursa; velum ~0.9 mm long, ~0.14 mm wide at widest point, 0.04 mm wide near middle. Spermatheca doughnut shaped, 0.38 mm in diameter, 0.16 mm deep, with invagination in center, 0.19 mm in diameter, 0.11 mm deep. Spermathecal duct very faint. Bursa copulatrix folded, pleated, small, barely covering dorsum of spermatheca, with distal end tapered, attached directly to sclerotized spermathecal velum, without bursal duct; bursal glands not found. Colleterial complex, transverse sclerite not discerned.

##### Variation.

Unknown.

##### Known distribution.

Central America. Belize (Cayo), Costa Rica (San Jose), Honduras (Colón, prob.), Nicaragua (Rio San Juan), Panama (Chiriquí).

#### ﻿ Santocellus helene sp. nov.

Taxon classificationAnimaliaNeuropteraChrysopidae

927EBA54-65FE-5FD7-AD4A-E4B18288A116

https://zoobank.org/C7E425AD-CFEB-431E-B5F7-CF28196281C5

**Figure 8.**
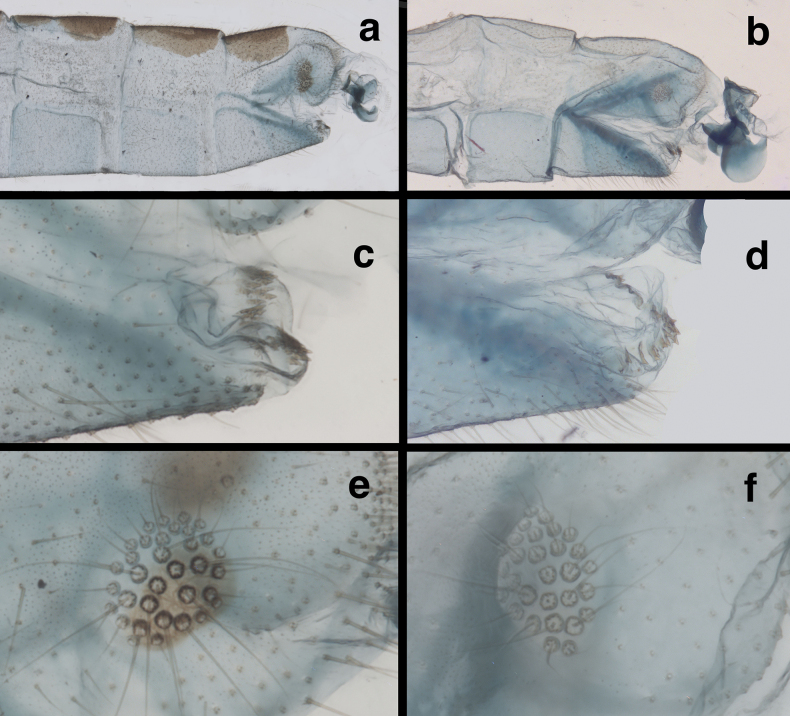
Side-by-side comparisons of male external abdominal features **a, c, e***Santocelluslegrandi* sp. nov., holotype, Nicaragua, Río San Juan, Bartola (CAS) **b, d, f***Santocellushelene* sp. nov., holotype, Bolivia, Cochabamba, Alto Palmar (CAS) **a, b** abdominal segments A6-terminus, lateral **c, d** terminus of S8+9, with terminal membrane bearing distinctive patches of gonocristae **e, f** callus cerci.

**Figure 9.**
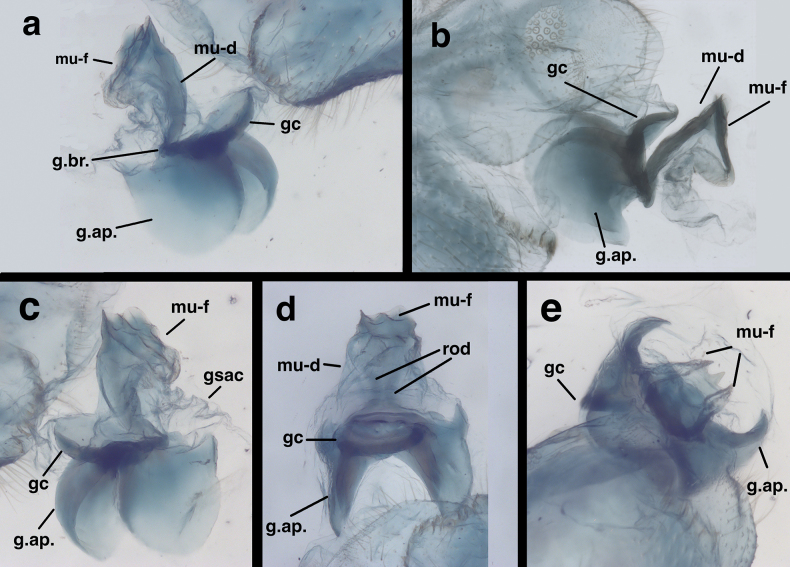
Gonarcal complex of *Santocellushelene* sp. nov., holotype, Bolivia, Cochabamba, Alto Palmar (CAS) **a** posterolateral **b** lateral **c** frontolateral **d** posteroventral **e** dorsal. Abbreviations: gc gonocornu (fused mesally, forming rounded plate); gsac gonosaccus; gst gonosetae; g.ap. gonarcal apodeme, dorsal view; g.br. gonarcal bridge; mu-d mediuncus, dorsal view; mu-f mediuncus, frontal view; rod internal mediuncal apodemes.

**Figure 10.**
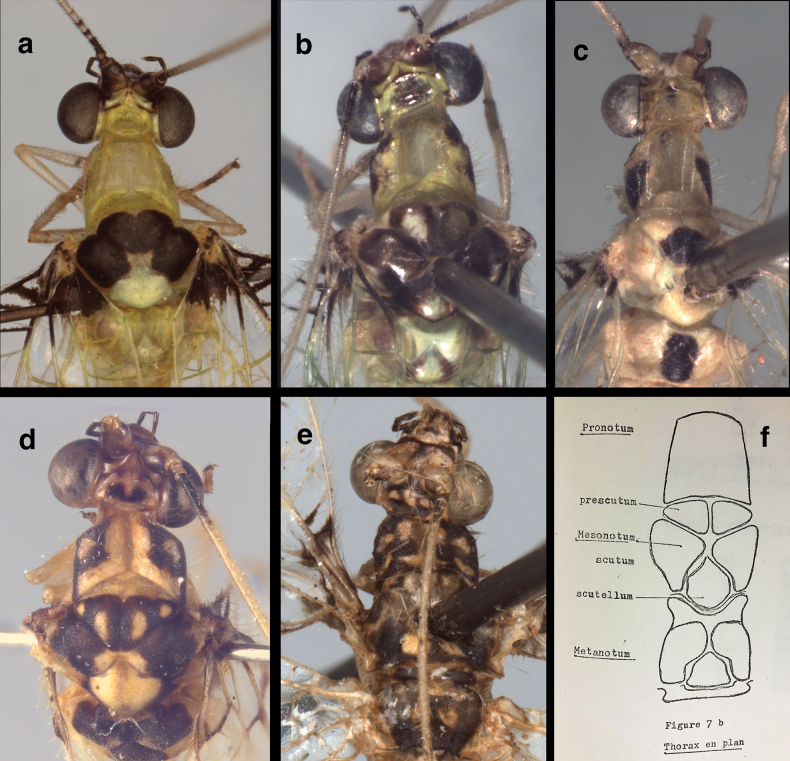
Side-by-side comparison of head and body (dorsum) of currently known *Santocellus* species **a***S.atlanticis*, male; Brasil, Rio Grande do Sul **b***S.riodoce*, female; Brasil, Espírito Santo **c***S.risi*, male; Madre de Dios, Peru **d***S.legrandi* sp. nov., male; Nicaragua, Río San Juan, Bartola **e***S.helene* sp. nov., male; Bolivia, Cochabamba **f** sketch of thoracic sclerites (from Séméria 1976).

**Figure 11.**
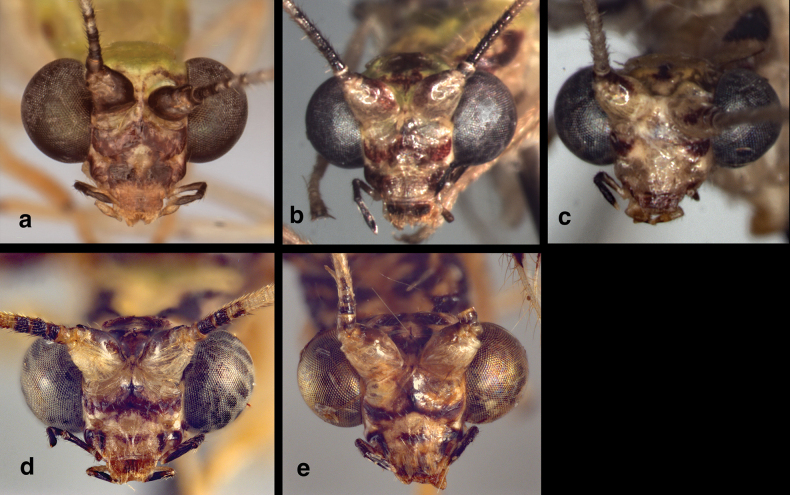
Side-by-side comparison of the head (frontal) of currently known *Santocellus* species **a***S.atlanticis*, male; Brasil, Rio Grande do Sul **b***S.riodoce*, female; Brasil, Espírito Santo **c***S.risi*, male; Peru, Madre de Dios **d***S.legrandi* sp. nov., male; Nicaragua, Río San Juan, Bartola **e***S.helene* sp. nov., male; Bolivia, Cochabamba.

**Figure 12.**
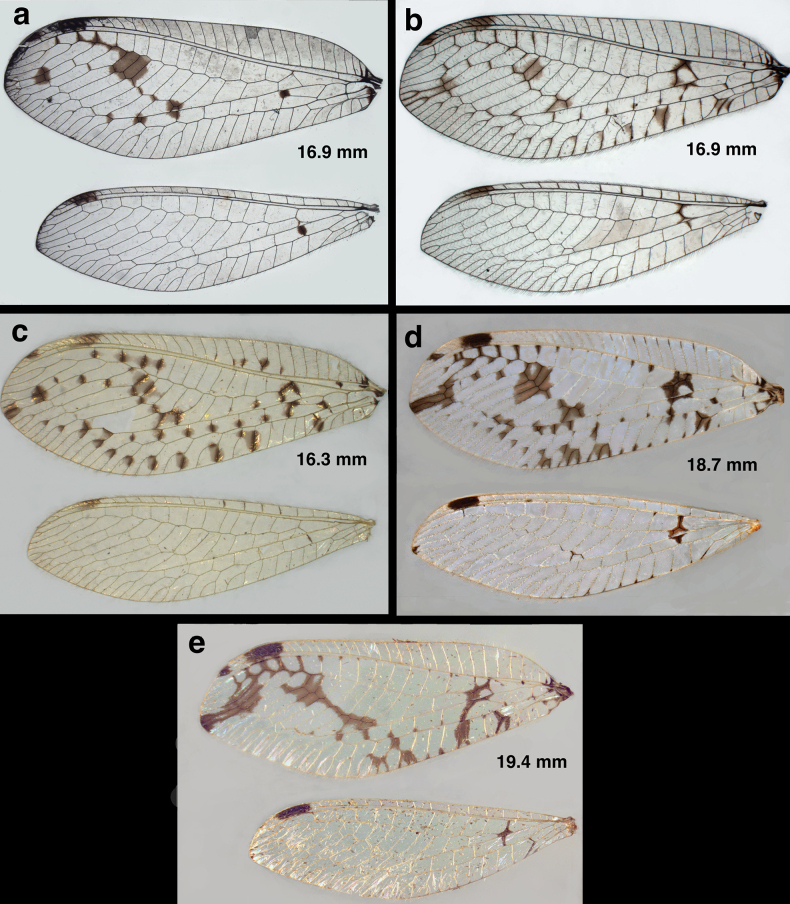
Side-by-side comparison of the wings of currently known *Santocellus* species **a***S.atlanticis*, male; Brasil, Rio Grande do Sul **b***S.riodoce* female; Brasil, Espírito Santo **c***S.risi*, male; Peru, Madre de Dios **d***S.legrandi* sp. nov., male; Nicaragua, Río San Juan, Bartola **e***Santocellushelene* sp. nov., male; Bolivia, Cochabamba. The numbers on the right of each image indicate the maximum length of the forewing recorded for that species.

Figs 3b , 7 , 8b, d, f , 9 , 10e , 11e , 12e

##### Etymology of name.

The species is named in honor of Hélène Legrand, wife of Jean Legrand (see Dedication above). The species name is her given name in apposition (restrictive) to the masculine genus name.

##### Type specimen.

**(sex and figures; verbatim label data [specimen location]) *Holotype*** • Male (Figs 3b, 7–9) – (1) Bolivia / Cochabamba / Alto Palmar, XI-60 – (2) Bought / F. H. Walz – (3) Phillip A. Adams / Collection / 1998 bequest to / Calif. Acad. Sci. – (4) *Gonzaga* n. sp.; – (5) Photos 4-23-79 [CAS]. No other specimens.

##### Description (based only on male holotype).

**Adult.** Medium-sized. ***Head*** (Fig. 7a–d): 1.81 mm wide (dorsum, including eyes); ratio of head width to eye width = 1.94:1; sutures not visible. Vertex raised, with small posterior fold. Antenna 23.6 mm long (~1.2 times length of forewing); scapes prominent, approximately as long as broad (0.17–0.18 mm). Pedicel round, tubular, not modified. Torulus relatively large, extending well below mid-eye level. Frons short to moderate length, with pale setae, lower margin scalloped upward above clypeus (below toruli). Tentorial pits distinct, round, raised. Gena 0.21 mm long; ratio of genal length to distance between tentorial pits = 0.47:1. Clypeus quadrate, except dorsal margin broadly curved upward below frons, distal margin relatively straight, pale mesally; surface rounded, slightly raised mesally, with sparse, amber setae of medium length. Labrum tapering anteriorly, with bilobed frontal margin, distinct cleft between lobes moderately deep, lateral margins tapering, long setae basally, shorter setae distally. Labium with submentum slender, rounded ventrally, with round, slightly bulbous tip. ***Coloration***: Scapes with frontal surface pale, mesal tip with dark mark extending onto mesal base of pedicel; pedicel tan throughout; flagellum largely pale, with basal three segments, distal segments dark brown to black. Lateral margins of vertex with pair of dark brown, curvy longitudinal bands, forming cream-colored circular spot centrally, each band with pale spot adjacent to circular spot, thus forming transverse row of three round cream-colored spots. Frons heavily marked with dark brown to reddish black distally, with broad, dark brown, scalloped band along margin above clypeus, laterally extending almost to mesal margins of eyes, mesally extending very short distance between scapes; area below scapes cream-colored, without markings. Clypeus with slender stripe across midsection, not reaching lateral margins; labrum with slight brownish tinge mesally. Clypeus with dark brown marks laterally, blending into dark distal marks on gena. Venter of head pale, pair of small brown spots near base of mentum and eyes; distal three maxillary palpomeres dark brown to black, basal two probably pale; distal labial palpomere black, basal two pale.

***Thorax*** (Fig. 7a, b, e): Prothorax approximately two times wider than long: 0.64 mm long, 0.31mm wide; ratio of length to width = 0.48:1. Surface with fine, golden setae, most short, some long to very long. Legs slender, with short to medium length, robust, setae, few long setae; tips of tarsi smooth, shiny (black) dorsally, with pair of smooth, small, swellings at terminus (Fig. 7e). Tarsal claw moderately long, narrow, base without dilation but with small mesal spur, two pairs of very long setae distally. ***Coloration***: [Note: cream- or tan-colored areas described here probably green in life.] Three thoracic segments mostly dark brown; pronotum with margins dark brown to black, center cream-colored, mottled with ~six large dark brown to black patches; venter, pleural region cream to light tan. Mesonotum, metanotum with paired cream and dark brown patches interspersed in roundish pattern. Legs pale, mostly unmarked, with setae mostly golden, few longer setae pale; surface of terminal segment (including base of tarsal claws and pair of small terminal swellings) shiny, black (Fig. 7e). Tarsal claw moderately long, narrow, with small spur; base slightly enlarged, without distinct dilation.

***Wings*** (Fig. 3b): Forewing (left) 19.4 mm long, 7.2 mm wide (maximum). Costal area moderately tall; tallest costal cell (#10) 1.7 mm tall, 0.24 times maximum height of wing. First intramedian cell triangular, stout, 1.2 mm wide, ~0.6 times width of third median cell. Origin of first radial crossvein ~0.32 mm distal to origin of Rs; radial area (between R and Rs) with single row of 14 closed cells; tallest radial cell 2.9 times taller than wide. Three b cells, each full sized, slightly longer than tall; first b cell heavily marked on all sides. Two series of gradates; 8 inner gradates, 9 outer gradates; size of cells bounded by gradates fairly uniform, lengthening slightly distally. Four b’ cells (beneath Psm, after im2); last b’ cell wider than tall. Three intracubital cells, distal one open; first cubital crossvein located basal to second mediocubital crossvein; icu1, icu2 each shorter than icu3; icu1+icu2 longer than icu3. Vein 1A forked. Terminal margin with approximately 5 simple veinlets, 13 forked veinlets.

Hindwing: 17.2 mm long, 5.2 mm wide; 12 radial cells (counted from origin of R, not false origin); t cell absent; first b cell beneath RP relatively small, quadrate, heavily marked. Two series of gradates; 6 inner gradates, 8 outer gradates. Three b cells; 4 b’ cells beyond im2; 3 intracubital cells, distal one open. Terminal margin with 5 simple basal veinlets, 12 forked basal veinlets. ***Coloration***: Wings hyaline, with bold dark brown markings. Forewing: stigma with large dark-brown spot basally, second smaller dark-brown spot distally; veins pale except in marks; penultimate sections of R, Rs, including R–Rs crossveins dark brown, surrounded by dark brown suffusion; slightly elongate dark brown mark at tip of wing, dark streak across center of wing, through inner gradate veins; dark-brown streak from base of Rs to margin of wing; scattered dark-brown marks along lower basal half of wing edge. Hindwing: stigma with one large dark-brown mark; base of RP, t cell absent; first b cell small, quadrate with dark-brown mark; distal section of cu (on posterior margin of wing) dark brown. Terminal margin with 4 simple veinlets, ~14 forked veinlets.

***Male abdomen*** (Figs 8, 9, slightly teneral). Segments 1–3 similar in size, proportion to distal segments, only slightly larger than basal segments. Fifth, sixth tergites (T5, T6) slightly taller than seventh tergite (T7). Tergites, sclerites, pleural region with numerous, short to medium length setae, microsetae; spiracles not enlarged; microtholi absent. Abdominal sternites moderately tall (S6: ratio of length to width = 1.2:1), with dorsal margins straight, slanting; distal sternites with slightly long, slender setae throughout. Callus cerci almost round to slightly ovate, 0.23–0.16 mm diameter, with ~31 long trichobothria; cupuliform bases of variable size [those within central section larger (~0.028 mm) than those on periphery (~0.015 mm)]. Dorsum of T9+ectoproct rounded distally, fused mesally with very small to no cleft; no midline suture visible; with long setae, densest along distal margin; dorsal apodeme straight, extending along full length of T9+ectoproct, above upper side of callus cerci, with large ventral spur extending downward along basal margin of callus cerci to posterodistal margin of ectoproct. S8+9 fused, apparently without suture, tapering to acute apex (lateral view); S9 slightly darker than S8; ventral apodeme extending across dorsal surface of S8+9; S9 spatulate distally, with terminal membrane prominent, attached across full margin of sternite; membrane bearing single, irregular row of large robust gonocristae laterally, smaller, denser gonocristae mesally.

***Male genitalia*.** Tignum, gonapsis, parameres, quadrate hood, pseudopenis, spinellae absent. Gonarcus broad, with gonarcal bridge bearing pair of elongate gonocornua fused laterally forming dome-like plate above mediuncus. Gonarcal arms large, rounded, expanded slightly forward. Mediuncus elongate, in two sections: basal section extending from below gonarcal bridge, with pair of elongate, rod-like internal apodemes covered dorsally by heavy subanal membrane; distal section of mediuncus extending distally from basal section at acute angle, with pair of flange-like arms lateral to mesal beak; gonosaccus beneath mediuncus, stiff, flat laterally, with few gonosetae.

***Female abdomen and genitalia*.** Unknown.

##### Variation.

Unknown.

##### Known distribution.

South America. Bolivia (Cochabamba).

### ﻿Species diagnoses

The two newly described species of *Santocellus* can readily be distinguished from other *Santocellus* species by their distinctive external markings on the body and wings (Figs 1–3, 7, 10–12). For example, both *S.legrandi* and *S.helene*, but not the other described *Santocellus* species, have forewings with large, heavy central markings and a pair of distal markings that extend in a band to the wingtip. The *S.legrandi* forewing markings differ from those of *S.helene* in that the two large central marks of its wings are separated from each other, and the apical marking extends only through the distal outer gradate vein; it does not reach the radial sector. In contrast, on the *S.helene* forewing the large central marks are connected, forming a contiguous, slender, slanted mark, and the apical marking extends from the wingtip well through the distal outer gradate veins and reaches the radial sector.

In addition to their distinctive external features, the males of the two species described here have diagnostic internal and genitalic features (Figs 4, 5, 8–10). Thus, it is prudent, when possible, to examine these features for accurate species identification and verification. For example, on *S.legrandi* the ventral margin of the terminal segment, T9+ectoproct, is deeply cleft (visible in lateral view), and the dorsal apodeme of T9+e curves slightly upward along the proximal margin of the callus cerci, but not beyond it (Fig. 8a). In contrast, on *S.helene*, the ventral margin of the terminal segment, T9+e, is straight; the dorsal apodeme extends above and beyond the callus cerci in a straight trajectory; and a ventral spur from the dorsal apodeme curves along and well below the proximal margin of the callus cerci. The gonocristae on the two species also differ: those on *S.legrandi* are numerous, moderately heavy, and broadly distributed in an irregular band across the dorsal surface of the terminal membrane of sternite 9, whereas those on *S.helene* are larger, less dense, and more robust; they also are positioned in a more defined row along the exterior margin of the terminal membrane.

### ﻿Updated species catalog of the genus *Santocellus*

*Santocellus* Tauber & Albuquerque, 2008

*Santocellusatlanticis* Tauber & Albuquerque, 2008, type species of genus.

*Santocellushelene* sp. nov.

*Santocelluslegrandi* sp. nov.

*Santocellusriodoce* (Tauber, 2007)

*Leucochrysariodoce* Tauber, 2007

*Santocellusrisi* (Esben-Petersen, 1933)

*Leucochrysarisi* Esben-Petersen, 1933

*Santocellusbullata* (Tauber, 2007)

*Leucochrysabullata* Tauber, 2007

### ﻿Key to *Santocellus* species

The key below is intended for species identification without dissecting specimens. Selected external features of all currently described species can be compared in Figs 10–12. However, as noted above, for reliable identifications male and female terminalia should be examined; illustrations of described abdominal and genital features are listed below.

**Figure 13.**
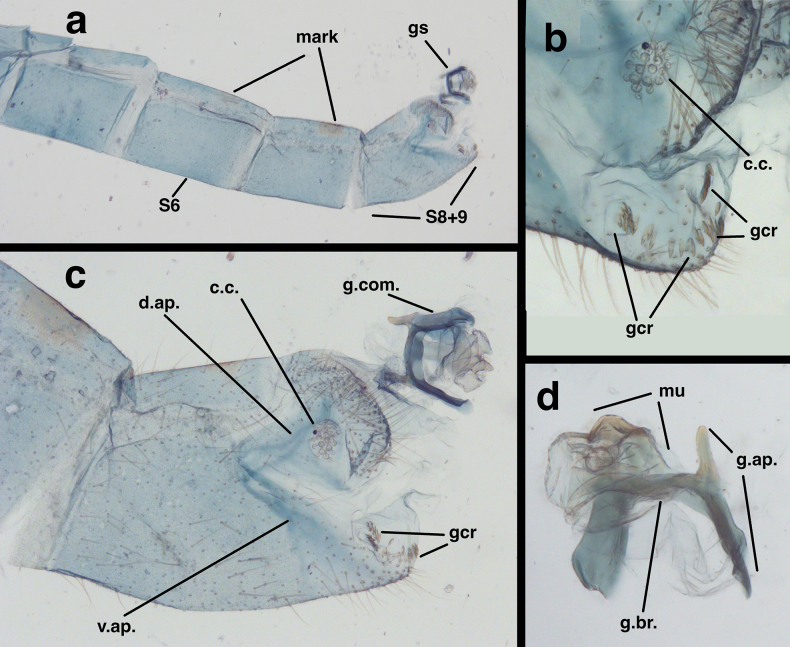
Previously unpublished images of the male abdomen of *Santocellusrisi* (Esben-Petersen). Bolivia: Cochabamba, 2280 M, 24-XI-1949, Luis E. Pena-G. (CAS) **a** lateral view of abdominal segments A5-terminus **b** tip of terminal abdominal segment (S8+9) bearing patches of gonocristae in two raised lateral patches and along distal margin of segment (**c**) terminal segments of abdomen (T8, T9+ectoproct, S8+9) length of T8 along dorsal margin, 0.68 mm **d** Posterior view of gonarcal complex. Abbreviations: c.c. callus cerci; d.ap. dorsal apodeme of T9+ectoproct; gcr field of gonocristae on terminal membrane of S9; gs gonarcus; g.ap. gonarcal apodeme; g.br. gonarcal bridge; g.com. gonarcal complex; mark brown mark on tergite; mu mediuncus; S6 sixth sternite; S8+9 fused eighth and ninth sternites; v.ap. ventral apodeme of S8+9.

**Figure 14.**
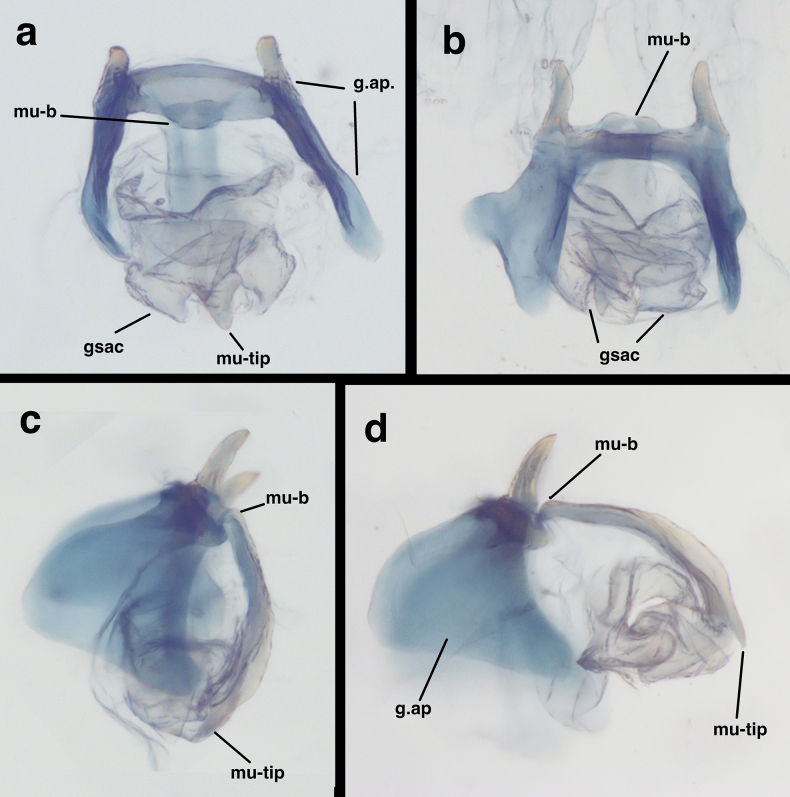
Previously unpublished images of the male genitalia of *Santocellusrisi* (Esben-Petersen). Bolivia: Cochabamba, 2280M, 26-XI-1949, L. E. Pena-G. (CAS) **a** gonarcal complex, frontal **b** gonarcal complex, posterior **c** gonarcal complex, lateral, with mediuncus folded below gonarcal bridge **d** gonarcal complex, lateral, with mediuncus extended outward from gonarcal bridge. Abbreviations: gsac gonosaccus; g.ap; gonarcal apodeme; mu-b base of mediuncus; mu-tip tip of mediuncus;. mediuncal membrane mu-tip tip of mediuncus.

*S.atlanticis*. Drawings: male and female: Tauber et al. (2008): figs 3–6. *S.riodoce*. Drawings: male and female: Tauber (2007): figs 21, 22, as *Leucochrysa*). *S.risi*. Drawings: male (as *Leucochrysabullata*): Tauber (2007): fig. 18; female: Tauber (2012): figs 2, 3; Figs 13, 14 (herein).

*S.legrandi* and *S.helene*. Images: male and female: Figs 4–6 and 8–10 (herein), respectively.

**Table:** 

1	Mesonotum and metanotum mostly pale, each with large dark brown to black mesal spot (Fig. 10c); surface of forewing shiny, with pustulate, swollen areas surrounding many crossveins (Fig. 12c)	***S.risi* (Esben-Petersen)**
–	Mesonotum largely or entirely dark brown to black, with or without light green (pale) areas/spots (Fig. 10a, b, d, e); surface of forewing smooth, not particularly shiny, without swellings (Fig. 12a, b, d, e)	**2**
2	Dorsum of head green, without markings except for stripe along posterior margin of upper antennal sulcus (Fig. 10a); forewing with fifth and sixth inner gradate cells entirely filled with brown; all crossveins between RP and PsM without dark clouding (Fig. 12a)	***S.atlanticis* Tauber & Albuquerque**
–	Dorsum of head with dark mark(s) centrally (Fig. 10b, d, e); forewing with fifth and sixth inner gradate cells only partially filled with brown; first two crossveins between RP and PsM with dark clouding (Fig. 12b)	**3**
3	Mesonotum distinctively darker than metanotum; dorsum of head mostly green, with reddish-brown spot mesally (Fig. 10b)	***S.riodoce* Tauber**
–	Mesonotum and metanotum both darkly marked; dorsum of head with dark, curved mesal marks (Fig. 10 d, e)	**4**
4	Pronotum darkly marked laterally, pale mesally (Fig. 10d); forewing with marking around inner gradate crossveins elongate, spanning approximately eight gradate cells (Fig. 12d)	***S.legrandi* sp. nov.**
–	Pronotum with dark markings throughout (Fig. 10e); forewing with marking around inner gradate veins short, spanning approximately four gradate cells (Fig. 12e)	***S.helene* sp. nov.**

## ﻿Conclusion

Previous studies distinguished the Neotropical genus *Santocellus* from other leucochrysine genera on the basis of a substantial suite of adult and larval traits (Tauber et al. 2008; Tauber 2012). However, from these same studies it was clear that additional comparative studies were necessary to confirm which of the traits truly typify all or most species within the genus and which are variable within the genus or among genera. With the additions here, the number of species described in the genus *Santocellus* grows to five – all from Central America and northern South America – and available information on the features that characterize the genus, or that vary within it, becomes better documented and accessible to comparative study.

## Supplementary Material

XML Treatment for Santocellus legrandi

XML Treatment for Santocellus helene

## ﻿Additional information

### Author ORCIDs

Catherine A. Tauber https://orcid.org/0000-0003-1310-8996

### Data availability

All of the data that support the findings of this study are available in the main text.
